# The necessity of suction drainage when intra-articular injection of tranexamic acid is used during primary total knee arthroplasty: a retrospective study

**DOI:** 10.1186/s12891-024-07604-w

**Published:** 2024-06-18

**Authors:** Yukie Metoki, Dai Iwase, Yasuaki Kusumoto, Jun Aikawa, Manabu Mukai, Kensuke Fukushima, Gen Inoue, Masashi Takaso

**Affiliations:** 1 Department of Orthopedic Surgery, Kitasato University School of Medicine, 1-15-1 Kitasato, Minami-Ku, Sagamihara City, Kanagawa 252-0374 Japan; 2 Department of Physical Therapy, Fukushima Medical University School of Health Sciences, 10-6 Sakaemachi, Fukushima City, Fukushima 960-8516 Japan

**Keywords:** Total knee arthroplasty, Suction drainage, Tranexamic acid, Intra-articular injection

## Abstract

**Background:**

Suction drainages are commonly used after total knee arthroplasty (TKA) procedures; however, their use is somewhat controversial. Recently, some reports have claimed that the administration of tranexamic acid (TXA) may prevent postoperative bleeding following TKAs. Although numerous studies have reported regarding different dosages, timings of administration, or drain clamping times for intravenous and intra-articular TXA injections (IA-TXAs), few have examined whether suction drainage is necessary when TXA is administered. In this study, we compared using suction drainage without TXA administration and IA-TXA without suction drainage and aimed to examine the need for suction drainage during IA-TXA.

**Methods:**

This retrospective study was conducted on 217 patients who had received TKA for osteoarthritis; 104 were placed on suction drainage after TKA without TXA (Group A), whereas the remaining 113 received IA-TXA immediately after surgery without suction drainage (Group B). Our clinical evaluation included assessments of the need for transfusion, presence of postoperative complications, incidence of deep vein thrombosis (DVT), and changes in hemoglobin (Hb), hematocrit (Hct), and D-dimer levels.

**Results:**

No significant differences were observed in terms of postoperative complications and preoperative Hb, Hct, or D-dimer levels between the two groups. Although the prevalence of DVT was significantly higher in Group B (*p* < 0.05), all cases were asymptomatic. Hb and Hct levels were significantly lower in Group A than in Group B at 1, 3, 7, and 14 days postoperatively (*p* < 0.05), although none of the cases required blood transfusions. D-dimer levels were significantly higher in Group A than in Group B at 1 and 3 days postoperatively (*p* < 0.05).

**Conclusion:**

Suction drainage might not be necessary when IA-TXA is administered after TKA procedures.

**Supplementary Information:**

The online version contains supplementary material available at 10.1186/s12891-024-07604-w.

## Background

Total knee arthroplasty (TKA) is an effective orthopaedic surgical procedure for improving pain and function of the knee joint, with a number of reports in the literature having described its favorable outcomes [1–3]. However, TKA occasionally causes postoperative complications such as bleeding, deep vein thrombosis (DVT), and infection [4]. Although suction drainage is commonly used following this procedure, its use remains controversial. This is because suction drainage during TKA results in more blood loss, as the drainage interferes with the tamponade effect [5], and because it results in a higher risk of secondary infection caused by retrograde bacterial transfer [6]. Over the last decade, some studies have reported that the use of tranexamic acid (TXA) may prevent postoperative bleeding after TKA, thereby reducing bleeding and the need for blood transfusions. In those reports, the administration methods included intravenous, intra-articular injection (IA), peri-articular injection, and oral route—all of which have been investigated in terms of dosage and timing of administration [7–9]. However, more of these reports indicated that suction drainage is used postoperatively, and only a few discussed the need for suction drainage using IA-TXA. Therefore, in this study, we compared using suction drainage without TXA administration and IA-TXA without suction drainage and aimed to examine the need for suction drainage during IA-TXA.

## Methods

In this retrospective study, we analyzed data from a total of 371 patients (423 knees) who underwent primary TKA at a single institution between January 2014 and December 2018. For perioperative management, suction drainage was used without TXA administration from January 2014 to December 2015, and IA-TXA was used without suction drainage from January 2016 to December 2018. The inclusion criterion was primary TKA to treat osteoarthritis, performed by one surgeon (D.I.), in a setting where autologous blood storage could be performed. Autologous blood storage was performed 3 weeks (400 mL) before surgery and was for patients with hemoglobin (Hb) levels ≥ 11 g/dL. The exclusion criteria included the undergoing dialysis; autoimmune diseases such as rheumatoid arthritis; preoperative anticoagulant use; prostheses with options such as extension stems, augmentation blocks, and semi-constrained prostheses; bilateral primary TKA performed on the same day; no autologous blood storage; surgeries performed by other doctors; and discharge from hospital within 13 days postoperatively.

**Fig. 1:**
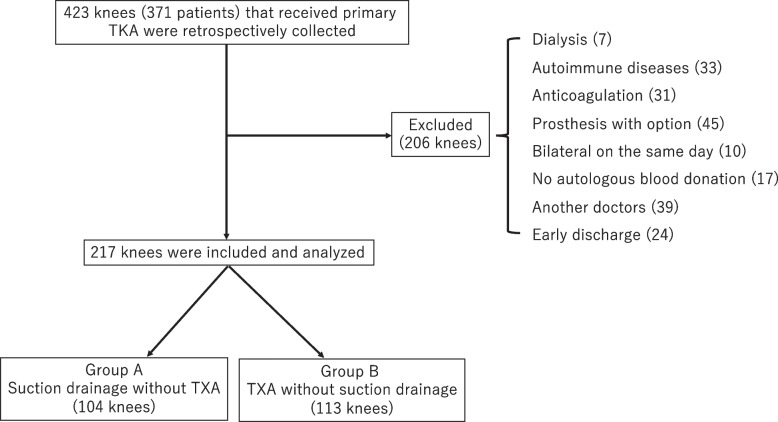
Flow diagram presenting the number of patients included in the study. Finally, 217 patients were included in our analysis. TKA, total knee arthroplasty; TXA, tranexamic acid

Ultimately, 217 knees were included in the study (Fig. 1), which were then categorized based on their perioperative management into Group A (104 knees), for which suction drainage without TXA was used, and Group B (113 knees), which received IA-TXA immediately after surgery, without suction drainage.

### Surgical procedures and perioperative care

In all cases, an air tourniquet was used during surgery, and a medial parapatellar approach was followed. Both femoral and tibial components were fixed using the cemented technique. The prostheses used were Persona® (Zimmer Biomet, Warsaw, IN, USA) or Scorpio NRG® (Stryker Orthopaedics, Mahwah, NJ, USA). In Group A, suction drainage—which allows for autologous transfusion using a blood conservation system—was routinely performed at the time of closure without TXA. The drains were not clamped and were removed on the second postoperative day. The drained blood was reinfused within 6 h of surgery. The mean drainage volume was 876.7 mL (range, 360 − 1820 mL), and approximately 60% of drained blood was reinfused (mean, 522.2 mL; range, 160 − 1040 mL). In Group B, TXA (2,000 mg) with saline (20 mL) was injected intra-articularly after surgery without suction drainage. The dose of IA-TXA was set at 2000 mg based on a report by Digas et al. [10]. Autologous blood transfusion (ABT; 400 mL) was performed intraoperatively or immediately after surgery for all patients. Enoxaparin (2000 IU) was administered subcutaneously twice daily (total 4000 IU) for 2 weeks as postoperative thromboprophylaxis. For patients weighing < 50 kg or with creatinine clearance < 50 mL/min, enoxaparin (2000 IU) was administered only once daily. During postoperative rehabilitation, the patients in Group A began range of motion and standing training immediately after drain removal, whereas those in Group B began range of motion and standing training on the first postoperative day.

### Outcome measures

Demographic features (including age, sex, and body mass index [BMI]) prior to surgery, and operative time were noted in all cases. Regarding laboratory data, Hb, hematocrit (Hct), and D-dimer levels were evaluated preoperatively (before autologous blood storage) and at 1, 3, 7, and 14 days postoperatively. Blood transfusion status, postoperative complications, and the occurrence of distal and proximal DVTs were also investigated. DVTs were evaluated using ultrasonography on postoperative day 7.

### Statistical analysis

Normality of the parameters was confirmed using histograms. The two groups were compared using the unpaired Student’s t-test for age, BMI, and operative time and Chi-squared test for sex, postoperative complications, and postoperative DVT. The postoperative course of these parameters was examined using repeated-measures tow-way analysis of variance and multiple comparison testing (via the Bonferroni method). Statistical analyses were performed using IBM SPSS Statistics for Windows, ver. 27 (IBM Corp., Armonk, Tokyo, Japan). A *p-*value of < 0.05 was considered statistically significant.

## Results

No significant differences were observed in terms of age, sex, BMI, or postoperative complications between the two groups. Operating time was significantly longer in Group A. The prevalence of DVT was significantly higher in Group B (*p* = 0.02). The incidences of postoperative DVT, none, distal, and proximal, were 79.8%, 19.2%, and 1.0%, respectively, in Group A and 63.7%, 31.9%, and 4.4%, in Group B. However, no cases were symptomatic. No allogenic blood transfusions were needed in either group. Hb, Hct, and D-dimer levels had a main effect between groups and on the postoperative course, confirming an interaction effect. Preoperative Hb, Hct, and D-dimer levels did not differ significantly between the groups. Hb and Hct levels were significantly lower in Group A at all time points after the first postoperative day. D-dimer levels were significantly higher in Group A at 1 and 3 days postoperatively. In the comparison of postoperative course, both groups had significantly higher values in all time periods after the first postoperative day compared to the preoperative values (Table 1).

**Table 1:** Pre- and postoperative data of both groups

		Group A (*n* = 104)	Group B (*n* = 113)	*p* value
Age, years		73.8 ± 7.4	72.2 ± 7.0	0.10
Sex: male, female (%)		23, 81 (22.1, 77.9)	26, 87 (23.0, 77.0)	0.88
BMI, kg/m^2^		26.9 ± 4.5	27.4 ± 4.9	0.41
Operation time, min		125.2 ± 20.0	118.6 ± 20.1	0.02^*^
Post-op complications: + , -		2, 102	1, 112	0.47
Post-op DVT: none, distal, proximal		83, 20, 1 (79.8, 19.2, 1.0)	72, 36, 5 (63.7, 31.9, 4.4)	0.02^*^
Hemoglobin, g/dL	Pre-op	13.4 ± 1.4	13.5 ± 1.4	0.62
	POD 1	10.8 ± 1.3	11.8 ± 1.6	< 0.01^*^
	POD 3	10.0 ± 1.5	11.5 ± 1.3	< 0.01^*^
	POD 7	10.1 ± 1.4	11.3 ± 1.3	< 0.01^*^
	POD 14	10.7 ± 1.2	11.8 ± 1.4	< 0.01^*^
Hematocrit, %	Pre-op	40.4 ± 3.8	41.1 ± 4.0	0.26
	POD 1	32.3 ± 3.9	36.0 ± 3.8	< 0.01^*^
	POD 3	30.1 ± 4.4	34.9 ± 4.1	< 0.01^*^
	POD 7	30.5 ± 3.9	34.4 ± 3.9	< 0.01^*^
	POD 14	32.4 ± 4.1	36.1 ± 4.1	< 0.01^*^
D-dimer, μg/mL	Pre-op	1.62 ± 1.83	1.80 ± 2.21	0.57
	POD 1	55.7 ± 76.2	6.8 ± 4.0	< 0.01^*^
	POD 3	6.4 ± 3.5	4.6 ± 1.7	< 0.01^*^
	POD 7	9.6 ± 3.9	9.8 ± 4.4	0.70
	POD 14	9.9 ± 4.0	11.1 ± 5.9	0.08

Group A: closed suction drainage without tranexamic acid, Group B: intra-articular tranexamic without closed suction drainage. Mean ± standard deviation

*BMI* Body mass index, *Post-op* Postoperative, *DVT* Deep vein thrombosis, *Pre-op* Preoperatively, *POD* Postoperative day

^*^p < 0.05

## Discussion

The primary aim of this study was to examine the need for suction drainage during IA-TXA. Although the prevalence of DVT was significantly higher in Group B, no cases were symptomatic. Group B exhibited significantly higher levels of Hb and Hct than Group A from postoperative day 1, whereas the D-dimer levels were significantly lower on postoperative day 1 and 3 days. There was also only one case of a postoperative complication, and no blood transfusions were needed in either group.

One of the major drawbacks of TKA is the perioperative bleeding and related complications caused by blood loss—with the estimated amount of bleeding after TKA varying between 800 and 1,800 mL [11]. Allogenic blood transfusions are required at variable rates among different reports, with one systematic review reporting that they were used in an average of 44% of patients [12]; however, various other measures have also been used to deal with hemorrhage. TXA administration has been widely used to control postoperative bleeding after TKA, and intravenous and IA-TXA have both been reported to significantly reduce blood loss and transfusion rates compared to no TXA administration [13–16]. Since approximately 10 years ago, there have been scattered reports of comparative studies on different methods of administering TXA. In three reports that compared intravenous TXA vs. IA-TXA, Sarzaeem et al. reported that intravenous TXA was more useful, Digas et al. noted that IA-TXA was more useful, whereas Patel et al. reported that they were equally effective [10, 17, 18]. Furthermore, Li et al. reported that IA-TXA was superior to intravenous TXA in terms of blood loss, drainage output, and Hb level on postoperative day 3 in their randomized controlled trial (RCT) that recommended IA-TXA for primary TKA [19]. In recent years, several reports have compared combined intravenous + IA-TXA with intravenous and IA-TXA alone [20, 21]. In 10 RCTs involving 1,306 patients, Ling et al. concluded that the combination of intravenous + IA-TXA was effective in reducing total blood loss, transfusion rate, postoperative Hb drop, and drainage output [20]. However, most of these reports used suction drainage postoperatively, and although there have been reports concerning suction drainage clamping time, there have been none to date that compared IA-TXA without suction drainage with no TXA administration with suction drainage [8, 9].

The use of suction drainage is long-existing common practice after TKA; however, a number of studies in the literature have questioned its necessity. Although the main purpose of suction drainage is to prevent hematoma, some reports claimed no difference without drainage in terms of blood loss, transfusion rate, knee joint range of motion, and postoperative complications [22, 23]. The American Academy of Orthopedic Surgeons 2015 clinical practice guidelines recommend not using suction drainage with TKA [24]. Moreover, the potential risk of retrograde infection with suction drainage has also been noted [25]. Hishimura et al. reported that a safe and effective alternative to suction drainage is needed as a hemostatic method [26]. In this study, ABT drainage was used instead of conventional closed suction drainage in Group A, and both groups also had a 400 mL autologous blood reservoir. Concerning the use of ABT, it reportedly decreased transfusion rates compared with closed suction drainage but showed no difference in terms of postoperative Hb drop [27, 28]. Although Tsukada et al. stated that there was no need for ABT even in single-anesthetic bilateral TKA [29], Tomura et al. reported that the perioperative Hb change was relatively small with ABT and the transfusion rate was 1%, suggesting that the cost-effectiveness of this method is debatable but nevertheless beneficial [30].

Regarding the incidence of DVT, Huang et al. reported that in a study of 92 TKA knees with intravenous TXA, ultrasonography was performed on postoperative days 1 and 3 and at discharge, and DVT occurred in 1.1% of patients [31]. However, in reports of TKA with TXA, ultrasound was performed only when DVT was suspected [10, 16, 18, 21, 32]. Ito et al. performed ultrasounds on postoperative day 7 in all patients in TKA without TXA administration and reported an overall incidence of DVT of 31.6% with a proximal incidence of 2.5% [33], similar to the results of the present study. Furthermore, all cases were asymptomatic, and no participant developed pulmonary embolism in this study.

Regarding D-dimer levels after TKA, Chotanaphuthi et al. noted that TKA resulted in severe trauma and bleeding, and with the application of bone cement, amongst other factors, the postoperative D-dimer levels rose rapidly and peaked at 24 h postoperatively [34]. Further, Helwig et al. suggested that D-dimer levels increased due to transfusion of recovered blood, as shed blood is deficient in coagulation factors and platelets and may contain increased levels of fibrin degradation products from the lysis of clots [35]. In contrast, Yang et al. reported that the increasing trend in D-dimer levels was inhibited after TXA, thus confirming that TXA inhibits early postoperative fibrinolysis and achieves hemostasis [36]. In our study, as in previous reports, D-dimer levels were significantly higher in Group A than in Group B on postoperative days 1 and 3.

We acknowledge that the present study had certain limitations. First, it was a retrospective single-setting study. Data from prospective randomized studies are more systematic and comprehensive, and the results of multi-center studies are more convincing. Second, no patients in both groups were allogenic transfused, one reason that everyone performed 400 ml autologous blood strage. In the future, studying the situation without autologous blood storage is deemed necessary. Third, this study did not examine the suction drainage group with IA-TXA. If this group had been included in the study, we might have been able to further assess the need for suction drainage more clearly. Regardless of these limitations, we believe that our study enabled us to draw meaningful conclusions.

## Conclusions

The present results showed that IA-TXA without suction drainage was equivalent to using suction drainage without TXA administration. Although these findings do not conclusively suggest that IA-TXA could be an alternative to suction drainage, suction drainage might not be necessary when IA-TXA is administered after TKA procedures.

### Supplementary Information

Supplementary Material 1.

## Data Availability

Data is provided within the manuscript and supplementary information files.

## Abbreviations

TKA: Total knee arthroplasty
DVT: Deep vein thrombosis
TXA: Tranexamic acid
IA-TXA: Intra-articular injection of tranexamic acid
Hb: Hemoglobin
ABT: Autologous blood transfusion
BMI: Body mass index
Hct: Hematocrit
RCTs: Randomized controlled trials
