# Supplementary material for: The necessity of suction drainage when intra-articular injection of tranexamic acid is used during primary total knee arthroplasty: a retrospective study
Source: BMC Musculoskelet Disord. 2024 Jun 18;25:475. doi: 10.1186/s12891-024-07604-w (PMC11184754; doi:10.1186/s12891-024-07604-w)
Supplement: Supplementary file 1 — Supplementary Material 1. [file 12891_2024_7604_MOESM1_ESM.pdf]

	0:–, 1:+	0:–, 1:+		0:M, 1:F			0:–, 1:+
case	drainage	TXA	age	sex	BMI	Ope time(min)	complication
1	1	0	71	1	26.6	125	0
2	1	0	69	1	25.9	110	0
3	1	0	75	0	18.9	97	0
4	1	0	87	1	25.9	110	0
5	1	0	66	1	22.6	140	0
6	1	0	76	1	26.6	85	0
7	1	0	73	1	19.3	135	0
8	1	0	70	0	25.8	120	0
9	1	0	82	0	28.9	115	0
10	1	0	76	1	27.8	123	0
11	1	0	64	1	22.7	135	0
12	1	0	70	1	29.9	140	0
13	1	0	78	0	27.8	150	0
14	1	0	73	1	20.7	105	0
15	1	0	76	1	26.4	135	0
16	1	0	66	1	26.8	135	0
17	1	0	76	1	23.7	105	0
18	1	0	65	1	22.8	125	0
19	1	0	80	1	31.4	119	0
20	1	0	71	0	23.4	130	0
21	1	0	70	1	28.0	116	0
22	1	0	78	1	24.2	170	0
23	1	0	62	1	29.7	125	0
24	1	0	82	1	32.1	125	0
25	1	0	77	1	30.7	129	0
26	1	0	86	1	21.7	100	0
27	1	0	71	1	22.0	98	1
28	1	0	72	1	22.4	133	0
29	1	0	72	1	26.5	121	0
30	1	0	71	1	22.5	99	0
31	1	0	60	1	41.5	216	0
32	1	0	62	1	25.1	100	0
33	1	0	75	1	28.8	125	0
34	1	0	81	1	25.0	119	0
35	1	0	66	0	31.4	130	0
36	1	0	73	0	32.9	136	0
37	1	0	82	0	26.4	143	0
38	1	0	50	0	32.6	114	0
39	1	0	74	1	25.2	90	0
40	1	0	80	1	26.9	104	0
41	1	0	78	1	25.4	125	0
42	1	0	82	1	24.1	117	0
43	1	0	67	1	27.5	117	0
44	1	0	79	1	22.4	123	0
45	1	0	58	1	32.6	110	0
46	1	0	86	0	24.9	119	0

47	1	0	63	1	30.9	138	0
48	1	0	80	1	25.0	138	0
49	1	0	71	1	21.7	115	0
50	1	0	75	1	26.5	129	0
51	1	0	72	1	26.6	144	0
52	1	0	76	1	18.6	106	0
53	1	0	76	0	29.8	110	0
54	1	0	66	1	30.0	101	0
55	1	0	69	1	20.0	125	0
56	1	0	77	1	29.5	118	0
57	1	0	77	1	30.8	130	0
58	1	0	83	1	24.1	134	0
59	1	0	77	1	28.3	103	0
60	1	0	81	1	30.5	133	0
61	1	0	82	1	36.2	109	0
62	1	0	74	1	34.7	157	0
63	1	0	79	0	25.6	112	0
64	1	0	67	0	26.3	125	0
65	1	0	73	0	24.7	141	0
66	1	0	78	1	31.2	118	0
67	1	0	72	0	26.0	113	0
68	1	0	72	1	20.4	113	0
69	1	0	82	1	24.9	115	0
70	1	0	63	1	29.8	109	0
71	1	0	76	0	27.8	148	0
72	1	0	65	1	29.4	101	0
73	1	0	77	1	35.8	138	0
74	1	0	78	1	25.0	130	0
75	1	0	87	0	24.8	100	0
76	1	0	80	1	23.6	138	0
77	1	0	61	0	22.6	150	0
78	1	0	77	1	22.0	135	0
79	1	0	63	1	24.1	129	0
80	1	0	74	1	30.8	130	0
81	1	0	87	0	22.4	92	0
82	1	0	80	1	31.8	140	1
83	1	0	62	1	44.8	134	0
84	1	0	77	1	26.1	160	0
85	1	0	63	1	24.2	135	0
86	1	0	83	0	25.1	114	0
87	1	0	81	1	22.2	135	0
88	1	0	69	0	29.3	173	0
89	1	0	78	1	31.6	128	0
90	1	0	75	1	22.9	138	0
91	1	0	82	1	22.4	139	0
92	1	0	71	1	27.4	125	0
93	1	0	73	1	25.9	128	0
94	1	0	76	1	37.1	109	0

95	1	0	72	1	28.7	149	0
96	1	0	77	0	30.1	118	0
97	1	0	80	1	29.9	110	0
98	1	0	72	1	20.0	87	0
99	1	0	52	1	28.5	140	0
100	1	0	81	1	22.3	141	0
101	1	0	68	1	30.7	114	0
102	1	0	77	0	27.3	181	0
103	1	0	70	1	26.1	134	0
104	1	0	85	1	26.0	118	0
105	0	1	74	1	36.2	129	0
106	0	1	78	1	30.7	123	0
107	0	1	77	1	26.4	130	0
108	0	1	76	1	21.0	99	0
109	0	1	70	1	29.4	133	0
110	0	1	62	1	31.1	126	0
111	0	1	72	1	20.5	114	0
112	0	1	72	1	24.9	92	0
113	0	1	74	1	28.6	136	0
114	0	1	72	1	35.6	139	0
115	0	1	78	0	24.7	105	0
116	0	1	72	1	23.4	140	0
117	0	1	71	1	26.3	107	0
118	0	1	77	0	28.6	123	0
119	0	1	73	1	17.9	162	0
120	0	1	72	1	35.2	123	0
121	0	1	70	0	29.4	139	0
122	0	1	65	1	28.5	119	0
123	0	1	72	1	28.8	109	0
124	0	1	81	1	29.9	102	0
125	0	1	75	0	23.6	217	0
126	0	1	85	1	23.4	143	1
127	0	1	76	1	22.9	109	0
128	0	1	75	1	25.7	102	0
129	0	1	72	1	22.4	128	0
130	0	1	68	1	22.5	121	0
131	0	1	68	0	22.5	107	0
132	0	1	76	1	23.9	134	0
133	0	1	72	1	22.1	133	0
134	0	1	73	1	18.7	118	0
135	0	1	74	1	25.9	97	0
136	0	1	61	1	34.8	122	0
137	0	1	78	1	24.1	119	0
138	0	1	80	0	28.7	123	0
139	0	1	72	0	27.8	103	0
140	0	1	75	1	26.4	146	0
141	0	1	79	0	28.6	119	0
142	0	1	76	1	25.5	110	0

143	0	1	69	1	19.8	124	0
144	0	1	63	1	31.1	132	0
145	0	1	69	1	22.4	134	0
146	0	1	63	1	29.9	129	0
147	0	1	72	0	29.8	110	0
148	0	1	65	1	33.3	97	0
149	0	1	75	1	26.2	124	0
150	0	1	84	0	23.9	133	0
151	0	1	74	0	26.8	117	0
152	0	1	82	1	32.5	143	0
153	0	1	83	1	29.6	121	0
154	0	1	61	0	27.1	122	0
155	0	1	79	1	29.6	144	0
156	0	1	79	1	29.3	144	0
157	0	1	69	1	27.1	130	0
158	0	1	76	1	30.9	126	0
159	0	1	79	1	25.1	119	0
160	0	1	69	1	29.4	139	0
161	0	1	76	1	26.4	123	0
162	0	1	76	0	25.7	95	0
163	0	1	74	1	26.1	200	0
164	0	1	70	1	22.7	97	0
165	0	1	73	1	24.3	120	0
166	0	1	75	1	31.4	128	0
167	0	1	67	1	28.8	144	0
168	0	1	62	1	34.8	94	0
169	0	1	63	1	33.2	103	0
170	0	1	75	0	28.9	129	0
171	0	1	74	1	32.8	122	0
172	0	1	65	1	39.2	127	0
173	0	1	74	0	26.1	126	0
174	0	1	70	1	19.8	99	0
175	0	1	73	1	23.3	103	0
176	0	1	75	1	25.4	130	0
177	0	1	85	0	26.0	101	0
178	0	1	73	1	43.5	99	0
179	0	1	48	0	31.6	107	0
180	0	1	70	0	31.8	103	0
181	0	1	81	1	22.5	138	0
182	0	1	91	0	26.7	117	0
183	0	1	77	1	31.4	107	0
184	0	1	74	0	26.7	117	0
185	0	1	73	0	25.1	110	0
186	0	1	77	1	22.5	121	0
187	0	1	70	1	24.5	114	0
188	0	1	71	1	31.9	114	0
189	0	1	78	1	24.2	81	0
190	0	1	60	0	23.5	85	0

191	0	1	76	0	32.4	113	0
192	0	1	57	1	28.1	108	0
193	0	1	62	1	36.5	121	0
194	0	1	73	1	25.7	95	0
195	0	1	71	1	21.8	96	0
196	0	1	58	1	31.9	81	0
197	0	1	74	1	20.4	96	0
198	0	1	74	1	22.8	127	0
199	0	1	66	1	24.5	103	0
200	0	1	74	1	25.9	130	0
201	0	1	73	1	20.0	92	0
202	0	1	78	1	31.3	106	0
203	0	1	67	1	26.0	135	0
204	0	1	74	1	27.6	102	0
205	0	1	64	0	33.4	128	0
206	0	1	75	1	20.7	113	0
207	0	1	66	1	27.8	93	0
208	0	1	80	0	28.1	107	0
209	0	1	71	0	40.9	133	0
210	0	1	43	1	39.9	140	0
211	0	1	76	1	19.9	109	0
212	0	1	77	1	24.1	110	0
213	0	1	71	1	27.6	95	0
214	0	1	69	1	24.1	100	0
215	0	1	60	1	26.2	120	0
216	0	1	70	1	35.8	99	0
217	0	1	81	1	24.4	106	0

	DVT(0:none, 1:distal, 2:proximal)	Hb				
case	Post DVT	pre Hb	1d Hb	3d Hb	7d Hb	14d Hb
1	0	13.2	10.3	8.8	8.5	8.4
2	0	13.6	11.9	11.6	11.3	12.1
3	0	16.7	11.9	9	11.3	12.6
4	0	11.9	8.2	7.4	8.8	9
5	0	12.6	10.4	8.9	10.2	11
6	0	13.2	11.4	8.5	9.1	10.1
7	0	12.2	11.1	9.8	9.3	11
8	0	14.4	11.2	9.3	9	10.1
9	0	14.1	12.4	12.9	11.9	12.5
10	1	14.8	12.4	11.5	10.6	10.9
11	0	17.1	13.4	11.7	10.9	12.3
12	0	14.7	11.7	10.6	10.1	11.4
13	0	14.9	10.8	9.4	9.6	11
14	0	13.5	10	9.2	9.3	11
15	1	14	11.4	9.8	9.7	11
16	1	13.6	11.4	10.7	11.1	11.6
17	1	12.2	10.3	8.7	9.4	10.7
18	0	13.2	11	10.8	10.5	12.4
19	0	10.6	10	9.3	9.2	9.6
20	0	13.9	12.2	11	10	11.1
21	0	12.8	11.4	10.4	11.1	11.1
22	0	12.7	9.3	7.4	7.2	8.2
23	0	15.5	12.2	12	12.3	11.9
24	0	10.4	7.8	7.2	7.8	7.4
25	1	15.1	13.4	12.8	10	10.1
26	1	12.3	9.7	8.5	9.8	9.3
27	1	13.8	12	11.8	13.3	11.6
28	0	13.4	10.4	8.9	8.4	9.8
29	0	10.8	8.5	8	7.8	8.6
30	0	14	12.9	12.8	12.1	13
31	0	15.4	11.3	12	11.3	11.1
32	0	13.3	10.4	10.6	10.5	11.2
33	0	12.8	9.6	8.5	8.6	9.2
34	0	12.4	10.1	9.1	8.5	8.5
35	0	14.5	12.3	12	12.5	12.6
36	0	14.8	13	13	13.2	12.9
37	0	11.8	9.5	10.1	10	9.8
38	0	15.4	12.8	11.2	10.2	11.4
39	1	12.6	11.9	11.1	11.8	11.3
40	0	12.3	10.9	10.1	11.5	11.5
41	1	13	11.4	9.9	10.8	10.8
42	0	12.3	10	8.8	8.8	10.3
43	0	14.1	10.9	11	10.9	11.5
44	0	13.8	10.8	11.3	11.1	11.3
45	0	13.7	10.7	8.6	8.5	9.7
46	0	14.5	13.2	12.2	11.8	11.9

47	0	15.2	11	9.6	9.6	9.6
48	1	12.9	9.2	7.5	8.2	8.9
49	0	13	10.8	11	11.4	12
50	0	13.2	9.8	8.6	9.9	10.4
51	0	12.9	9.2	9.2	8.3	10.4
52	0	12.6	9	10.7	10	10.8
53	0	13.2	11.4	9.9	10.8	10.1
54	0	13.1	10.3	9.9	10.3	10.6
55	1	11.3	8.8	8.3	9.2	9.5
56	0	11.1	10.1	10.9	9.6	10.7
57	0	13.3	10.7	9.7	9.9	11.2
58	0	11.8	9.6	8.5	8.1	9.8
59	1	14.6	10.9	10.3	10.2	11
60	0	10.4	9.5	8.9	9.1	9.8
61	0	13.7	12.2	11.3	9.9	11
62	0	12	10	10.4	9.9	9.7
63	0	17.1	12.5	11.6	11.9	11.6
64	0	14.9	12.7	12	13.1	12
65	1	12.4	9.8	9.9	10.1	11.1
66	1	14.2	12.4	12.2	12.4	12.9
67	0	14.4	12.3	11.9	12.1	12
68	0	14.1	11	11.1	11.6	11.1
69	1	13.5	9.7	9.4	9.8	10.1
70	0	13.7	9.8	9.5	9.8	10.9
71	0	14	11	10.6	9.9	11
72	0	12.9	9.8	8.8	8.8	10
73	0	13	11.6	10.4	10.1	10.5
74	2	13.7	9.8	8.5	8.1	8.4
75	0	14.9	11.7	10.2	10.3	10.1
76	1	12.2	8.5	7	9.3	9.3
77	0	14.4	10.8	10.6	11.1	11.3
78	1	14.3	10.1	9.8	9.3	11.8
79	0	10.2	8.7	8.5	8.8	10.6
80	0	16	13.1	12.2	12.7	11.9
81	1	15.4	10	7.8	8.1	8.7
82	0	11.3	8.3	7.4	8.3	9.8
83	0	15.4	14.2	11.6	10.8	13.6
84	0	14.6	9.3	8.8	9.9	9.8
85	0	14.2	12.9	11.7	11	11.4
86	0	14.3	11.9	13.1	11.8	12.6
87	0	12.4	10.2	9	9	9.3
88	0	13.5	9.1	8.2	8.2	9.7
89	0	11.3	11.1	9.3	10.2	10.3
90	0	11.3	9.5	9.3	9.4	10.8
91	0	13.6	12.2	13	11.5	12
92	0	13	9.5	9.5	9.4	9.6
93	0	13.4	11.5	9.9	10.7	10.7
94	0	13.3	10.3	7	9	9.7

95	0	13.7	9.5	8.8	8.8	9.9
96	0	11.9	9.8	9.7	9	9.4
97	0	11.5	10	9.8	9.4	10.5
98	0	13.9	12.1	11.9	12.6	11.8
99	0	13.1	11.5	9.9	9.5	10.4
100	0	11.4	8.9	8.5	8.4	8.4
101	1	13.5	10.2	9.2	9.1	9.7
102	0	13.7	11.3	11	10.7	11.8
103	1	13.5	11.3	10.3	9.2	10.4
104	0	11.9	9	9	9.5	10.3
105	0	11.3	10.5	10.8	11.3	10.7
106	2	11.1	10.8	11.2	11.5	11.8
107	0	12	10.4	11	9.8	10.2
108	1	10.7	9.3	8.4	9.3	9.4
109	0	14.4	12.8	12.7	12.6	13.4
110	0	13.5	11.1	10.5	10.5	10.5
111	0	15.1	11.1	12	12.3	12
112	0	12.8	11.1	11	11.4	11.6
113	1	14.2	11.6	12	11.9	12.4
114	0	14.7	11.5	11.8	11.2	11.8
115	0	14.6	12.7	12.4	11	11.7
116	1	13.1	11.7	10.7	10.9	11.7
117	0	14.4	13.5	12.5	12.9	12.8
118	0	12.9	12.5	11	10.8	12.5
119	1	13.8	12.1	11.5	11	11.2
120	0	12.1	10.9	10.2	10.1	10.5
121	0	15.2	11.9	11.2	11.9	11.4
122	1	13.8	12.1	12.1	10.8	11.4
123	0	12.6	10.7	11.6	11.3	11.6
124	0	13.5	11.7	11.6	12	12.2
125	0	15.3	11.4	9.9	9.1	9.8
126	1	11.5	10.3	9.5	9.1	10.6
127	0	14.5	12.1	11.9	11.6	11.8
128	0	13.6	10.8	10.6	9.2	10.7
129	0	13.7	13.4	11.9	11.3	10.1
130	1	12.4	12	12	11.3	11.8
131	0	16	14.7	14.1	13.8	14.2
132	1	11.6	11	10.7	10.3	10.2
133	0	13	11	10	9.3	9.6
134	0	12	12	11	10.5	11.3
135	2	14.2	12.5	13	12.3	12.8
136	0	15.5	12.9	11.7	12.9	13.1
137	0	12.1	10.4	11	10.6	10.3
138	1	12.3	11.6	10.4	10.2	11
139	0	13.8	12.3	11.4	10.1	11.8
140	1	12.8	12.1	11.4	10.7	10.9
141	0	15.4	12.2	11.5	11.6	13.2
142	2	12.5	10	9	9.5	10.1

143	1	13.4	11.6	11	10.7	12
144	0	14	11	10.4	10.9	11.4
145	0	13.2	10.7	10.9	10.2	10.6
146	0	13.9	12.9	11.9	11.8	12.6
147	0	18.3	17.2	17.3	16.5	18.3
148	0	14.9	13.3	13.1	13.1	13
149	0	13	12.8	12.6	11.5	11
150	1	14.9	11.7	11.9	11.9	12.2
151	2	15.1	12.8	12.7	13.1	13
152	0	13.6	11.9	11.4	11.8	13.1
153	0	15.4	12.4	11.6	11.4	12
154	0	15.8	15.3	14.2	14.2	15.6
155	0	13	10.2	11.5	11.5	11.2
156	0	11.6	10.9	11.2	11.5	11.7
157	0	14.7	12.8	11.8	11.8	12.3
158	0	13.1	12.3	12.9	12.9	12.5
159	1	12.5	1.9	10.1	10.7	10.9
160	0	12.1	9.5	8.7	8.6	9.2
161	0	12.4	11.3	11.3	11.2	11.6
162	1	14.4	12	12.3	12.7	13.2
163	1	15.9	13.6	13.1	12.5	12.7
164	0	12.7	11.2	11.1	10.5	9.8
165	1	11.4	10	8.9	9.3	9.7
166	1	14.3	14.3	13.9	12.8	13.1
167	0	14.5	12.2	11.9	11.5	11.8
168	0	15.6	14	14.1	14.1	14.4
169	1	13	11	10.8	11.1	11
170	0	14.2	13.4	14.3	13.7	13.7
171	0	13.1	12.1	11.6	11.8	12.4
172	1	13.5	12.9	12.2	12.4	12.5
173	0	15.6	14.9	11.6	10.8	11.2
174	0	12.5	11.7	11.8	12.2	12.2
175	2	11.9	11.5	9.7	9.9	10.2
176	0	12.7	11.4	10.3	9.9	10.4
177	1	14.5	12.5	12.5	12.5	12.7
178	1	11.4	9.3	10.3	9.6	9.7
179	0	15.3	11.7	10.7	11.2	11.7
180	0	15.9	12.3	9.8	7.8	9.2
181	1	12.5	11.2	10	10.3	10.3
182	1	12.7	10.2	11.1	10.3	10.8
183	0	14.2	12.3	12.3	11.6	11.5
184	1	15.2	13.7	13.8	12.2	13.8
185	0	14.4	12.8	12	13.5	13.1
186	0	13.5	10.6	11.1	10.3	10.6
187	0	12.4	12.1	10.2	10.2	11.1
188	1	13.9	12.1	12	11.5	12.4
189	0	9.9	10.3	11	10.7	10.7
190	1	14	12.9	10.7	11.3	12.7

191	0	12.7	12.3	10.8	10.1	9.1
192	0	13.5	10.7	9.9	9.4	13
193	0	13.5	12.2	12	11.4	13.5
194	0	13.5	9.5	10.5	10	11.7
195	0	12.8	11.5	11.4	11.2	11.6
196	0	13.1	12.7	11.4	11.2	11.1
197	1	12.8	12.2	12.3	12.3	12
198	1	14	11.5	10	11	11.7
199	1	14.6	12.6	14.3	12.1	13.9
200	0	13.9	14.1	13.3	13.6	13.9
201	0	12.3	11.6	11.2	11.6	11.8
202	0	13.5	13.1	12.5	12.6	13
203	1	12.4	12.1	11.3	11.2	10.6
204	1	12.6	10.1	10.8	11.6	10.8
205	1	12.9	12.3	11.8	11.3	12.2
206	1	13.1	13.3	11.1	10.2	10.7
207	1	12.7	12.4	11.1	11.5	11.2
208	0	12.6	11.8	11.7	12.3	12.4
209	0	11.3	11.2	11.1	9.5	10.7
210	0	13.2	11.3	10.9	11.1	11.7
211	1	10.9	10.4	11	10.5	10.4
212	0	14.1	12.4	12.1	12.2	13.3
213	0	13.2	11.9	11.6	10.9	11.8
214	0	15.5	13.5	13.2	13	13.1
215	0	12.1	11.6	10.3	10.4	11.1
216	0	14.9	12.4	14.2	14	14.1
217	1	12.6	10.2	10	10.4	11.1

	Hct				
case	pre Hct	1d Hct	3d Hct	7d Hct	14d Hct
1	38.9	29.9	25.1	25.1	25.3
2	40.1	34.8	34.1	33.3	35.9
3	47.8	36.2	27.8	33.4	37.5
4	36.8	26.4	22.3	27	27.8
5	38.1	30.5	26	30.7	33.5
6	39.5	33.4	25.1	26.8	30.6
7	38	34.4	30.1	28.3	33.9
8	43.3	33.2	27.3	26.7	31.3
9	42	36.6	37.8	35.4	36.2
10	44.7	37.2	34.7	32.3	33
11	49.7	38.9	34.6	31.7	36.8
12	43.7	33.4	30.7	29.3	34.9
13	44	32.5	29.7	28.8	34.8
14	40	29.2	26.9	27.1	33.7
15	42.5	34.4	29.8	29	32.8
16	41	33.8	32.1	33.5	34.9
17	35.3	29	24.6	26.9	30.6
18	40.8	33.5	32.9	32.7	38.4
19	34.3	32.1	29.6	29.7	31.1
20	41	33.9	31.3	29	32.2
21	37.5	33.3	29.9	32.5	32.7
22	39.3	27.6	22.3	22.1	25.9
23	43.9	35	34.6	35.8	34.8
24	29.3	22.4	20.8	22.8	22.5
25	44.9	40.7	35.8	29.5	31.9
26	37.3	28.8	26	29.5	28.7
27	39.5	35.9	34.6	39.7	34.5
28	40.1	31.2	26.5	26.1	30.1
29	33.4	24.1	24.1	23.9	25.9
30	41.4	37.2	37.4	34.8	37.8
31	45.2	34.1	37.3	34.7	35.2
32	37.5	30.2	31.1	31.6	33.7
33	39.2	28.6	25.1	27.1	29.3
34	37.1	29.9	27.6	26.3	26
35	43.7	36.5	35.1	36.5	36.5
36	43.4	38.9	37.5	38	37
37	34.9	28.2	30.7	30	29.6
38	45.4	36.5	32.5	30.3	30
39	38.1	36.4	33.8	34.4	34.3
40	36.4	32.3	30.5	30.4	35
41	37.3	32.6	28.4	28.4	32
42	37.1	29	27.1	27.1	31.9
43	42.4	32.7	29.9	33.4	35
44	43	33.3	34.3	33.8	34.9
45	40.6	31.1	25.9	25.2	29.6
46	42.2	38.5	36.1	34.3	35.7

47	46.1	32.8	28.7	29.1	29.1
48	39.7	27.7	22.7	25.3	27.7
49	38.6	32	33.1	34.1	36.6
50	40	29.8	26.1	30	31.8
51	37.8	27.6	27.3	25.2	32.2
52	38.1	27.3	32.2	31.5	33.4
53	38.9	34.3	29.2	32.5	31.1
54	38.5	30.3	29.6	30	32.3
55	37.1	27.6	26.4	28.5	30.7
56	34	30.8	33.7	28.9	30.7
57	40.3	32.9	30.7	30.7	35.5
58	36.5	28.3	26.1	25.3	31
59	45.8	32.9	32.2	34.7	34.7
60	32.8	29.1	27.9	28.9	30.7
61	41.8	36.6	33.6	30.2	34.5
62	38.2	30.8	31.5	31.1	30.5
63	51.1	36.2	34.5	36.7	35.9
64	44.8	37.3	34.5	39.6	35.9
65	39.7	31	31	31.3	36.4
66	44.3	39	39	39.1	39.4
67	41.7	36.1	35.1	36	36.2
68	41.9	31.5	32.9	34.1	34
69	41	29.3	29	29.5	31.8
70	41.2	30.3	29.1	30.9	34.3
71	42.4	32.5	33.1	31	34.5
72	40	29.5	27.1	27	30.8
73	39.9	36.3	32.2	31.3	32.4
74	41.2	30.8	25	23.9	26.1
75	44.6	34	30	30.7	30
76	37.9	25.4	21.5	29.2	29.3
77	44.4	31.9	32.2	33.1	34.6
78	43.5	29.7	29.2	28.4	36.2
79	31.4	26.1	25.4	28	32.2
80	47.7	39.4	36.8	39.3	36.6
81	47	31.6	23.3	24.8	28
82	36.3	25.7	22.7	25.9	31
83	48.1	45.7	35.7	35.9	44.8
84	44.8	28.9	26.9	29.9	30
85	44.1	39.4	35.9	34.5	35.6
86	40.6	35.2	37.9	34.8	38.7
87	39.2	31.1	27.9	27.7	29
88	39.8	26.5	25.2	24.4	29.6
89	40.3	33.8	28	30.6	31.7
90	35.3	28.4	28.4	28.9	33.1
91	42.5	36.2	40.6	34.7	37
92	40.5	29.9	29.7	29.1	29.9
93	41	35.5	29.8	32.2	10.7
94	41.6	31.7	21.6	27.9	30.4

95	42.2	28.3	26.9	27.8	30.3
96	37.3	30	29.9	28.2	30
97	34.9	30.7	30.7	29	32.8
98	40.7	34.1	34.8	36.6	34.7
99	39.9	34.6	30.7	28.7	31.2
100	35.5	27.6	27.1	26.9	26.4
101	41.6	30.8	28.4	28.2	29.3
102	41	33.7	33.3	33	37.3
103	40.9	33.5	31.6	29.2	32
104	36.7	27.2	27.3	30.7	31.6
105	34.5	32.8	33	33.7	34.3
106	34.8	32.5	33.9	35.8	36.6
107	36.4	31.9	32.5	30.5	32.2
108	32.3	28.4	25.7	28.9	28.5
109	43.4	39.2	38.8	38.7	41.5
110	41.2	33.5	32	31.4	32.7
111	45.5	33.7	37.2	37.3	36.8
112	39.2	33.7	33.1	34.4	35.5
113	42.9	34.4	34.8	35.9	38
114	41.6	34.3	34.1	33.2	33.5
115	43.8	36.1	35.2	31	33.4
116	41.1	35.8	33.2	34.2	36.6
117	43.2	40	36.7	39.3	38.9
118	39.3	37.2	33	32.2	38.3
119	41.5	34.2	34.1	32.2	32.5
120	37.6	32.8	31.1	31.8	32.5
121	46.4	36.4	33.9	36.8	34.8
122	42.9	36.1	36.9	33.1	36
123	38.5	33.6	36.3	35.3	36
124	40.4	34.7	35.1	26.5	37.4
125	46.6	34.9	30	27.9	31.1
126	36.1	31.9	29.5	28.9	33.8
127	45.9	37.2	37	36	35.9
128	40.8	33.3	32.7	28.3	33.1
129	42.3	39.3	35.3	33.8	31.6
130	37.3	38	36.9	34.8	36.5
131	45.9	43.9	41.1	39.8	41.7
132	35.2	32.1	31.3	31	31.3
133	39.1	32.6	29.6	28.2	28.8
134	37.8	35.8	33.1	32.1	35.4
135	44	37.9	39.7	36.1	39.6
136	47.5	38.7	35.4	39.1	40.5
137	36.8	31.1	33	32.3	32
138	39.5	37.8	33	32.8	35.9
139	41.2	36.3	34.2	30.1	36.5
140	38.2	35.4	33.8	33.3	32.6
141	45.3	34.7	32.6	34.5	39.6
142	38.9	30.9	28	30.4	32.5

143	40.4	34.7	32.5	32.6	37.3
144	43.3	34.1	32.5	34	34.8
145	40.7	31.6	33.3	31.2	33.6
146	42.2	39	36.7	37.1	38.2
147	57.2	52.8	54.2	49.7	55.9
148	46.7	40.7	39.7	40	41
149	38.6	38.4	37.8	34.5	33.9
150	45.8	35.3	35.1	36.2	37.2
151	45.3	40.5	38.6	40.9	41.1
152	42.6	37.8	34.6	37	39.8
153	48.7	38.4	36	35.1	37.9
154	47.4	46.5	43	42.1	47.4
155	40.2	30.5	34.3	35	34.8
156	37.8	34.3	36.2	37.1	37.6
157	45.3	39.7	35.9	36.8	37.5
158	40.2	38.8	38.5	39	38.9
159	39.5	33.7	32.1	33.6	35.2
160	37.4	29	26.4	26.5	28.2
161	37.8	33.6	35	33.9	35.4
162	42.9	35.9	37.7	37.6	39.9
163	47.4	41.1	38.6	35.9	38.5
164	39.5	35.3	34.9	32.5	30
165	34.9	30.8	28.5	29.4	31
166	46.1	43.6	44.1	41.1	43.5
167	43.2	36.9	36	34.7	35.5
168	47.3	41.3	43.1	42.1	44.3
169	39.3	33.4	33.1	34.5	33.5
170	42.4	39.9	43.5	41.1	40.5
171	40.2	35.7	34.4	36.3	36.8
172	42.3	38.8	37.4	37.8	37.9
173	47.3	43.7	35.2	31.7	33.5
174	38.5	36	35.9	37.3	35.5
175	36.2	35.1	29.7	30.4	31.4
176	39	32.9	30.7	30.1	32.4
177	42	36.7	36	36	36.8
178	35.9	28.7	32.1	29.9	30.2
179	47.6	36.3	33.5	35.3	36.1
180	47.1	35.5	29.4	24.4	28.5
181	39.8	35.3	31.8	33	32.7
182	39.9	31.5	34	31.7	33.8
183	44	37.9	36.7	35.2	35.9
184	45.1	40.2	41.3	35.9	42.1
185	42	38.1	35.4	40.5	39.8
186	40.7	32.1	33.4	30.1	32.2
187	38.2	37.1	30.2	31.6	34.9
188	41.5	37.4	36.3	35.7	39
189	32.5	32.8	34.7	32.5	34.4
190	42.6	38.2	32.2	34.2	38.5

191	39	36.9	32.4	30.4	29.1
192	40.2	30.9	29.2	28.9	38
193	40.3	36.9	35.6	35	39.8
194	40.9	29.7	31.7	31.5	33.9
195	38.2	35.7	35.1	34.5	36.7
196	37.9	38.1	33.6	33	33.4
197	39.3	36.1	37.1	35.1	35.5
198	41.4	34	30.1	34.1	36.4
199	46.5	39.5	44.3	38.7	42.3
200	42.2	43.3	41.6	42.5	44.1
201	36.7	35.2	33.4	35.7	35.9
202	39.8	39.6	37.1	38.2	38.8
203	37.5	37.2	34.3	33.9	32.2
204	37	30.9	32.6	34.8	32.8
205	39.5	36.8	36.2	33.8	36.8
206	39.2	38.4	32.9	31	33.3
207	37.9	36.7	33.5	34.6	34.4
208	39.2	35.8	35.4	37	38.3
209	34.5	32.8	33.8	29.1	33.5
210	40.2	34.6	32.6	34	36.5
211	34.7	32.8	34.2	33.3	32.6
212	40.7	35.7	35.3	36.1	38.8
213	40.7	36.3	34.9	33.1	36.5
214	46.8	38.8	40.2	38.7	39.7
215	37.8	35.1	31.1	31	33.8
216	45.5	37.5	44.4	41.8	41.6
217	37.9	29.5	29	31	32.9

case	D-dimer pre D	1d D	3d D	7d D	14d D
1	0.57	55.28	5.04	6.69	3.7
2	1.36	19.86	4.91	7.32	10.58
3	1.2	93	3.58	6.38	3.76
4	1.58	149.7	9.18	14.42	15.41
5	1.57	149.5	15.42	10.28	8.51
6	1.16	49.58	7.13	10.84	12.42
7	0.77	45.28	5.8	7.12	5.62
8	2.73	10.04	4.99	9.13	11.65
9	15.18	16.79	4.59	6.29	8.21
10	0.73	104	4.83	9.89	15.34
11	1.05	52.76	4.59	7.84	11.25
12	0.83	81.89	12.82	14.99	16.65
13	0.65	45.62	3.38	4.92	12.25
14	1.56	10.65	2.38	2.61	4.8
15	1.7	78.03	7.7	14.76	19.1
16	0.75	36.07	5.74	8.59	9.29
17	0.81	33.81	14.25	7.02	7.02
18	2.4	46.46	7.86	11.6	7.92
19	3.51	47.36	4.72	7.95	10.75
20	0.73	15.06	3.45	5.72	7.83
21	0.78	67.16	3.62	6.58	7.12
22	1.19	44.65	6.13	8.38	7.95
23	0.74	95.77	4.38	8.36	12.21
24	2.04	33.1	6.42	8.88	8.72
25	0.87	19.06	5.92	6.86	4.3
26	0.75	30.54	5.51	7.99	8.36
27	0.4	33.18	3.69	6.94	16.94
28	2.72	105.7	25.21	14.88	10.21
29	1.52	52.89	9.23	7.7	8.5
30	1.63	8.24	2.1	5.47	7.81
31	1.25	19.26	8.9	11.59	8.85
32	0.71	36.72	2.98	7.49	5.51
33	1.21	65.88	7.31	14.42	10.9
34	1.59	55.87	7.72	7.19	7.4
35	0.67	32.52	3.99	11.34	15.21
36	1.54	19.49	12.91	10.3	7.63
37	3.21	52.43	6.13	6.46	8.45
38	0.57	12.18	4.78	6.91	6.91
39	0.95	82.97	5.41	7.78	9.19
40	0.8	25.28	4.04	7.53	8.99
41	1.121	43.76	6.35	9.48	13.08
42	0.69	93.68	8.34	12.55	10.19
43	4.12	42.92	7.27	14.06	17.33
44	3.57	74.22	13	13.67	6.68
45	0.67	752	1.75	2.84	6.13
46	0.71	30.13	3.23	4.5	7.83

47	0.72	12.77	3.88	11.81	11.81
48	1.27	75.27	3.97	9.42	9.43
49	0.56	31.07	2.72	4.23	2.57
50	0.82	5.24	4.42	9.31	8.11
51	1.78	38.08	9.99	13.48	10.33
52	1.82	55.78	6.91	8.75	9.51
53	1.27	51.8	8.37	8.75	7.8
54	0.67	15.66	3.16	5.16	3.32
55	8.76	80.47	13.38	14.05	10.65
56	4.42	34.12	7.26	10.29	9.45
57	3.8	42.19	8.49	19.22	19.22
58	0.79	19.72	3.93	8.43	5.85
59	1.39	78.7	6.83	9.5	5.37
60	2.17	54.86	3.76	9.61	9.95
61	0.74	83.67	5.04	8.35	9.77
62	1.59	42.74	3.31	5.64	5.3
63	0.69	21.54	4.24	6.46	5.31
64	2.76	35.17	3.59	11.03	14.14
65	0.76	42.37	3.14	4.85	7.78
66	1.08	29.81	5.06	10.56	5.62
67	0.88	22.12	2.44	5.61	10.62
68	0.76	42.86	4.41	6.19	5.24
69	1.92	58.09	12	12.68	12.41
70	0.61	37.75	4.31	7.19	6.75
71	0.87	84.87	6.02	15.67	14.9
72	4.48	82.88	8.57	14.05	17.26
73	0.64	10.35	8.34	8.13	7.8
74	0.85	113.2	7.39	14.85	13.96
75	1.51	18.41	3.97	7.55	9.59
76	2.48	28.57	6.45	11.21	12.52
77	0.6	40.43	2.97	6.14	13.66
78	0.99	43.37	9.17	14.98	13.14
79	5.75	29.71	9.32	9.34	9.2
80	0.64	51.61	3.61	6.87	5.68
81	4.1	77.83	4.99	10.31	14.54
82	2.66	37.87	5.69	8.74	10.46
83	0.81	37.56	2.19	4.95	6
84	1.23	69.58	6.95	18.81	12.39
85	1.08	103.6	4.49	4.89	7.21
86	1.16	57.65	5.25	5.26	4.42
87	1	54.35	12.5	9.88	10.85
88	0.84	66.39	8.64	20.41	17.47
89	1.07	49.07	9.36	13.43	12.07
90	0.77	58.29	10.43	11.51	10.73
91	0.81	36.33	5.44	7.19	6.63
92	1.03	181.9	13.54	23.32	18.53
93	2.01	31.47	4.02	10.2	10.2
94	1.47	18.77	6.15	19.22	14.16

95	1.15	14.41	3.37	5.9	6.39
96	0.82	23.73	5.97	8.72	12.36
97	1.59	11.74	3.69	5.71	7.98
98	1.21	89.13	3.86	13.5	7.35
99	1.52	2.55	3.25	8.43	10.96
100	0.85	86	5.65	6.86	3.47
101	0.53	26.68	5.57	12.86	12.28
102	0.99	40.32	5.79	16.03	20.41
103	0.77	20	10.07	11.75	15.8
104	1.02	15.83	5.48	7.05	6.49
105	2.03	5.22	2.55	8.53	7.6
106	1.11	2.35	3.16	6.18	3.46
107	1.22	2.01	2.36	6.51	10.34
108	1.82	5.87	3.95	13.31	6.11
109	0.76	5.31	3.56	8.14	8.94
110	2.33	10.29	6.53	17.3	29.12
111	0.63	6.54	3.83	9.26	12.59
112	0.49	6.81	3.58	7.49	4.52
113	0.8	10.37	14.78	33.84	15.78
114	2.78	3.9	5.07	11.45	13.47
115	4.98	8.63	3.24	14.93	14.93
116	0.71	7.21	4.36	8.81	8.67
117	1.01	4.43	4.12	11.49	10.83
118	1.27	3.26	3.67	9.24	13.2
119	1.58	14.92	6.33	12.63	13.04
120	1.88	5.71	6.9	11.79	8.28
121	2.25	4.52	3.25	8.14	4.8
122	1.1	7.26	5.14	0.53	5.55
123	1.07	16.3	9.5	17.4	16.71
124	1.41	2.28	7.01	6.51	9.02
125	1.3	8.71	4.86	13.44	21.53
126	3.43	3.51	5.16	13.26	16.41
127	0.64	2.1	5.3	12.55	10.37
128	0.74	10.12	4.49	16.72	25.36
129	1.34	5.22	4.16	8.39	8.39
130	1.35	4.79	4.62	7.85	15.69
131	1.32	12.23	7.32	7.05	7.3
132	1.22	10.42	5.54	9.19	6.58
133	0.62	1.3	3.35	11.65	20.16
134	0.86	2	2.19	2.17	5.91
135	1.32	1.29	2.25	2.9	2.35
136	1.15	8.5	3.14	5.04	5.48
137	1.79	4.17	4.55	7.99	10.8
138	1.08	3.01	3.78	7.2	10.89
139	1.35	13.35	3.46	8.37	8.37
140	1.67	4.69	5.61	10.37	18.53
141	0.81	9.49	5.54	10.24	11.22
142	0.85	8.02	2.24	3.93	3.57

143	1.18	9.01	4.64	7.98	8.15
144	2.5	6.11	7.13	22.34	30.34
145	0.89	13.22	4.51	11.57	14.61
146	1.4	5.46	3.29	7.87	9.53
147	0.6	7.9	4.5	7.89	9.75
148	0.75	3.29	3.43	8.02	12.56
149	0.88	7.05	3.7	7.6	8.19
150	0.9	10.31	5.8	10.04	10.01
151	3.05	7.53	4.41	14.73	11.65
152	1.81	5.16	4.15	10.66	8.49
153	1.23	7.61	5.4	13.74	10.19
154	0.8	3.22	5.17	6.77	11.72
155	3.01	9.22	7	18.2	17.13
156	1.13	2.05	4.19	10.88	7.91
157	0.91	5.4	3.02	7.68	8.34
158	2.58	5.49	4.07	12.85	11.95
159	2.94	3.68	3.92	5.7	7.42
160	1.25	14.89	5.01	21.11	40.26
161	2.11	6.03	4.46	13.25	19.79
162	0.72	1.81	2.69	6.6	7.41
163	0.8	5.97	7.33	9.49	15.12
164	1.89	4.09	5.46	8.83	11.37
165	2.54	11.57	5.93	2.73	12.76
166	1.14	2.08	3.96	5.69	5.69
167	0.78	6.58	4.18	12.19	9.64
168	0.9	3.47	2.42	4.96	4.54
169	0.82	17.15	4.73	6.69	10.58
170	1	2.81	2.77	5.03	7.43
171	0.9	2.24	2.99	7.95	12.8
172	0.94	12.33	3.91	9.09	8.34
173	3.06	2.43	4.54	7.72	13.7
174	1.1	6.52	2.93	6.78	6.78
175	1.14	5.64	3.5	5.51	3.91
176	1.05	7.84	9	11.55	23.2
177	1.88	10.76	4.81	11.97	18.99
178	4.14	5.62	5.23	13.07	16.93
179	0.64	7.05	3.65	3.94	1.96
180	0.71	3.68	4.08	5.81	8.41
181	9.19	7.89	4.09	11.35	12.06
182	10.41	6.92	3.94	8.4	15.15
183	9.52	3.5	4.8	10.15	7.43
184	1.05	5.37	4.76	6.3	12.41
185	4.45	4.8	5.96	13.1	20.87
186	2.05	6.9	4.07	5.3	6.87
187	1.35	10.81	6.58	14.14	10.49
188	0.8	6.13	5.29	10.71	8.5
189	1.03	7.68	5.22	11.52	10.25
190	0.64	25.77	4.9	6.65	8.48

191	0.6	4.66	2.83	8.06	12.96
192	3.35	6.48	3.29	7.18	7.18
193	0.5	4.56	1.86	3.25	3.25
194	0.6	9.05	5.39	11.9	7.07
195	1.82	9.31	5.55	10.52	10.62
196	0.71	4.05	5.97	10.29	13.32
197	1.6	4.28	4.19	7.83	9.56
198	2.53	5.71	4.18	10.53	11.81
199	1.08	4.01	4.17	6.78	11.09
200	2.27	6.34	4.5	11.77	12.72
201	1.4	8.33	3,2	6.42	6.12
202	0.88	2.04	.2.94	7.73	12.47
203	1.54	5.53	4.83	12.98	9.41
204	1.19	6.82	6.48	8.76	13.93
205	0.73	4.83	2.87	7.35	7.14
206	0.92	4.65	4.64	11.71	7.71
207	1.17	15.38	5.29	12.22	5.1
208	1.32	5.83	3.35	6.4	7.72
209	1.1	2.51	5.02	16.76	15.98
210	1.01	3.58	2.93	9.51	13.41
211	18	14.75	4.67	14.81	11.12
212	1.7	8.24	5.45	12.2	6.9
213	0.82	13.73	4.26	10.85	13.9
214	1.87	11	5.76	10.97	9.25
215	0.71	2.96	3.19	6.68	4.74
216	1.59	2.46	3.69	8.51	6.83
217	1.03	7.35	4.9	11.78	9.47
